# Spatial-Temporal Evolution and Driving Factors of the Coupling Coordination between Urbanization and Urban Resilience: A Case Study of the 167 Counties in Hebei Province

**DOI:** 10.3390/ijerph192013128

**Published:** 2022-10-12

**Authors:** Weihao Shi, Jian Tian, Aihemaiti Namaiti, Xiaoxu Xing

**Affiliations:** 1 School of Architecture, Tianjin University, Tianjin 300072, China; 2 School of Applied Economics, Renmin University of China, Beijing 100872, China

**Keywords:** coupling coordination, urbanization, urban resilience, driving factors, counties, Hebei Province

## Abstract

Urban resilience, as an important ability to deal with disasters in the process of urbanization, has been paid more and more attention as the result of the increasing risks that are caused by rapid urbanization. China is taking the county level as the basic unit to promote new-type urbanization and constructing resilient cities has become one of the development strategies. However, to achieve this strategy researchers need to analyze the interaction between county urbanization and urban resilience and its driving mechanism, which have been paid little attention. Therefore, this paper selected 167 counties in Hebei Province as the investigation subject. Based on the statistical data from 2010 to 2020, a comprehensive index system was developed to quantify the degree of coupling coordination between urbanization and urban resilience, and the spatial Durbin model was used to analyze the driving mechanism of it. The study shows that: Firstly, the urbanization level of counties rose year after year, with there being a geographical distribution that was “lower from southeast to northwest”. The level of urban resilience increased year after year, showing a geographical distribution that was “higher from south to north” and a “core-edge” feature that was localized. Secondly, the coupling coordination degree increased steadily, and the overall level changed from a basic imbalance to a mild imbalance. In space, it is bounded by “Pingquan City—Pingshan County”, which showed the distribution of “high in the east and low in the west, high in the center and low on the outskirts”. Thirdly, the coupling coordination degree has spatial spillover effect. Government financial expenditure, innovation level, industrial upgrading level and urban shape index all influence the coupling coordination degree positively, with a successively decreasing impact, while the urban compactness has significant negative impacts. This study indicates that the regional differences exist in the coupling coordination degree, and the counties in different development stages need to adopt different strategies to promote the coordinated development of urbanized and resilient cities. Inter-regional support is also necessary in this process. Meanwhile, it is necessary for the government to govern various urban elements, especially in terms of their urban form.

## 1. Introduction

Since the reform and opening up that took place, China has witnessed the greatest and quickest urbanization process in history, with the urbanization rate increasing from 17.92% in 1978 to 59.58% in 2018, which is almost 1.04% growth per year [1]. Rapid urbanization has brought in population concentrations and land expansions [2,3], which has increased various risks in the cities. According to statistics, the annual economic loss that is caused by public security incidents in Chinese cities exceeds 500 billion yuan [4], which not only reduces the security of the cities, but also severely restricts the sustainable and high-quality development process of the cities [5]. Being a significant frontier theory in public safety, urban resilience provides a solution for solving urban security risks systematically [6,7]. Promoting the process of resilient cities has become an important strategy for urban development. In 2022, the National Development and Reform Commission suggested that the country should promote the new-type urbanization with counties being the significant carrier of it, and they took “resilience” as one of the goals of new urban construction [8]. Therefore, incorporating urban resilience into the urbanization course and realizing the coordination of urbanization and urban resilience is critical for realizing the sustainability and security of China’s urbanization. In light of this, this paper focuses on the following questions: (1) How do urbanization and urban resilience interact? (2) How is urbanization and urban resilience coordinated at the county level in China? (3) What are the driving forces of the coupling coordination degree?

As an important component of the Beijing–Tianjin–Hebei (BTH) metropolitan area, Hebei Province is currently in the period of rapid urbanization, and its urbanization rate crossed the critical point of 50% in 2017 and reached 60.07% in 2020. However, due to the extensive urbanization mode and weak infrastructure, Hebei is faced with the contradiction between urbanization and urban risk, thus it is a typical region that demonstrates the mismatched development between urbanization and urban resilience. Therefore, taking the county level of Hebei Province as the research area, scientifically assessing the coupling development levels of urbanization and urban resilience and exploring the driving mechanism of them is an important research basis for promoting the matching of the urbanization process and urban resilience in China, and this can also make up for the gaps in the independent research between the urbanization system and the urban resilience system in the existing research. In light of this, based on statistics on the urban resilience and urbanization of 167 counties in Hebei Province from 2010 to 2020, we built a comprehensive indicator system for the county urbanization level (economy, population, spatial and social urbanization) and the urban resilience (economic, ecological, social and infrastructure resilience), and we calculated the coupling coordination degree of them. Finally, the driving forces behind the coupling coordination were explored using the spatial Durbin model.

The following paragraph explains how this article is structured. Section 2 provides an overview of the relevant research background and literature, and it builds a theoretical framework for urbanization and urban resilience, whereas Section 3 details the datasets, resources and indicator systems as well as the methodologies that are used in this work. Section 4 analyzes the spatial-temporal evolution and the development stages of the coupling coordination. Section 5 examines and explores the causes of the coordinated evolution and the interaction between urbanization and urban resilience and puts forward the corresponding policy recommendations. Finally, Section 6 summarizes our findings.

## 2. Literature Review and Theoretical Framework

### 2.1. Literature Review

Urbanization is a complicated procedure of the fundamental changes in the economic structure, social structure, production and lifestyle [9]. With China’s urbanization shifting from quantitative to qualitative, the measurement of the urbanization level has developed from a single indication to a comprehensive indicator system. The traditional form of urbanization is mainly represented by the population and industry agglomeration, as well as the pursuit of increasing the number and scale of the cities [10]. While extensive urbanization caused a great disturbance to the ecosystem. In 2014, China proposed a new urbanization strategy, whose primary component was the sustainability and coordination of numerous urban aspects. The change in the direction of urbanization has affected the measurement of the level of urbanization. The implementation of the new urbanization strategy has made the measure of urbanization about much more than just the scale of the city and its population. Using a single indicator measurement method is inadequate to comprehensively assess the degree of urbanization [3]. Existing studies mainly measure the level of urbanization from four aspects, including demographic, economy, space and society [11,12,13]. This internal logic can be understood as: population urbanization is the most important part; economic urbanization is the necessary condition; spatial urbanization is the representation of the population and economic urbanization in regional space, which is also the carrier of urbanization; whereas social urbanization transforms people’s ways of life, behavioral habits, and values that accompany this process.

The concept of urban resilience originated from the studies of ecology that were conducted by Holling, and gradually formed the mainstream measurement framework of the comprehensive evaluation system that is used in various fields such as entire social-ecological systems [14,15]. In the 1990s, the field of urban planning progressively introduced the idea of resilience [16,17], which was mostly used to investigate the sustainable evolution of urban systems following catastrophes like diseases, floods and climate changes [18,19,20]. As a result of this period, the focus of resilience research has grown from a single integrated system to a complex social-ecological system, with there being an emphasis on the system’s shift from an equilibrium to a dynamic non-equilibrium [21], and thus, the connotation of resilience was further enriched. According to the United Nations Agency for Disaster Risk Reduction (UNISDR) and the Rockefeller Foundation, urban resilience is the capacity of various systems such as groups and societies to withstand, absorb and adapt to consequences when they are facing a variety of long-term pressures and sudden shocks [22,23]. Although the resilience theory provides a novel approach for promoting urban sustainability, there is currently no agreement on how to quantify urban resilience. [24]. In recent years, scholars have developed various tools and models such as survey interviews [25], evaluation systems [26,27,28], socio-physical networks [29], landscape ecological models [30] and system simulation models [31] to measure the resilience of provinces, urban agglomerations and single cities from the view of urban landscapes’ ecology [30], infrastructure [32], communities [33] and level of disaster resilience [22]. The differences in the urban resilience evaluation systems stem from the use of different perspectives. However, it is certain that the studies of urban resilience are comprehensive, covering variety of topics. A city is a massive complicated system that is made up of its infrastructure, economy, society, ecology, etc. It is of great importance to assess the comprehensive resilience of it using a multi-system framework.

The relationship between urbanization and urban resilience is complex and dynamic. In general, the research of Kamila et al. [18] shows that urbanization has always been negatively related to urban disasters. According to the study of Suarez et al. [34] on Spanish cities with a high degree of urbanization, they tend to have a lower level of resilience than those cities with a lower urbanization rate. However, other research has shown that the impact of urbanization and urban resilience is indirect and complex, and the relationship between them is reflected in many aspects including the urban ecology, land and society. In terms of ecology, with rapid urbanization, the inherent properties of the cities such as ecosystem services are reduced, which are believed to help resist sudden changes, destruction and natural disasters [35]. Feng et al. [36] demonstrated from the perspective of landscape ecology that urban sprawl leads to a reduction in urban resilience. In terms of the urban space, a research study that was conducted on the Yangtze River Delta urban agglomeration revealed that there is a complicated link between land use and urban resilience in the urbanization process [37]. From the perspective of social development, Kamila et al. [18] compared the resilience of different countries, indicating that urbanization led to an increase in the population density in the urban areas, which worsened the impact of the disasters in these cities. In addition, the growth of informal settlements and the lack of social equity during urbanization [38] can also result in the reduction in social resilience. A city is a sophisticated system, and different cities (developed or developing; large or small) face various resource trade-offs in the process of urbanization [18,39], which leads to a complex relationship between urbanization and urban resilience.

Based on the complex relationship between urbanization and urban resilience, the driving factors that affect the coupling degree of urbanization and urban resilience are also diverse. According to the existing research, we abstractly categorize it into five levels: the natural elements, the spatial form, government governance, the economic level and the innovation ability. In terms of the natural factors, the urban geographical attribute is an important factor hindering the urban expansion, which has an impact on the urban population distribution [40]. Meanwhile, poor geological conditions will affect the occurrence of floods, earthquakes and other disasters [41], which will have a joint impact on urbanization and urban resilience. In terms of the spatial form, the process of urbanization has significantly changed the urban spatial structure and form, which has an impact on the resilience level. On the one hand, evidence shows that the urban spatial structure has a promoting effect on strengthening the level of urban resilience [42], on the other hand, the urban form has an important impact on land use efficiency [43], innovation and transformation, and environmental governance [44], thus indirectly affecting urban resilience. However, there are a few studies on urban resilience that are related to the urban form at present, and different conclusions have been produced. Therefore, the impact of the urban form on the coupling coordination degree is complex and it needs to be further expanded. In terms of government governance, the level of government governance and urbanization mutually reinforce one another, and the capacity of the local governments is critical to the establishment of urban resilience [18]. The government uses public financial expenditures to prevent disasters and reduce the disaster losses [45], while encouraging economic growth and environmental conservation [46], thus, it has a positive impact on the coordination degree between urbanization and urban resilience. In terms of economic development, urbanization has led to a shift in the use of economic development strategies to realize the diversification and advancement goals, and the diversified industries are necessary conditions for the resilient cities, therefore, the higher the degree of complexity of the industrial structure, then the stronger the city’s ability to resist external economic risks will be, and the stronger the relationship between urbanization and urban resilience will be. In terms of innovation, the core of the influence of the economic development factor on the coupling coordination degree is the potential innovation [18]. On the one hand, scientific and technological innovation promotes the transformation of urban industries, changes the way in which resources are used, and on the other hand, enhances the creativity of the labor force, which will have a positive impact on urbanization and urban resilience at the ecological, economic and social levels.

The studies on urbanization and urban resilience mostly adopt qualitative methods such as case study method [47], the literature review method [15] and correlation analysis [18]. Chelleri et al. [47], for example, explore the trade-offs of urban resilience in urbanization by analyzing the cases at three scales in the Netherlands, Bolivia and Kampala. There are also a small number of studies which focus on the relationship between urbanization and urban resilience from the perspective of coupling, such as that which was conducted by Gao et al. [48] who explored the coupling relationship between urban resilience and urbanization quality in 14 cities in Liaoning Province. However, the coupling relationship is mostly used to measure the interaction between two or more systems, and therefore, simple calculations cannot produce a thorough understanding of it. For the study of the coupling relationship between urbanization and urban resilience, there is a lack of a set of overall methodologies that range from theoretical construction to experimental research to mechanism exploration, which is indispensable for the promotion of the urbanization and urban resilience coordination relationship.

Overall, the existing research has had a positive impact on the research on urbanization and urban resilience. However, they have some disadvantages. Firstly, many scholars are aware of the dialectical relationship between urbanization and urban resilience, and they have made a qualitative exposition, but they have not specifically quantified the relationship and explored the mechanism of it. Secondly, the existing research is limited to the national, provincial and urban agglomeration scales. Although these is helpful for understanding coordinated regional development, it is obviously difficult to provide effective and accurate guidance for the development of counties in the course of the new-type urbanization with counties as the main body while using these scales. Therefore, the possible contributions of this paper are as follows: (1) In terms of the research areas, urban resilience is a new topic emerging in the context of China’s new urbanization, and this paper builds a theoretical framework and a quantitative analysis of urbanization and urban resilience, thereby providing new ideas for the government to accelerate the construction of resilient cities. (2) In terms of the research scale, we focus on the spatial analysis units at the county level, which is more implementable than previous studies are at the provincial and municipal levels. (3) In terms of the mechanism analysis, we discuss the potential impact mechanism of the coupling coordination from five perspectives, including government governance and the urban form.

### 2.2. The Theoretical Framework between Urban Resilience and Urbanization

In general, there is a remarkable link between urbanization and urban resilience. On the one hand, urbanization has different impacts on urban resilience in different periods. At the beginning of the urbanization process, the high concentration of a large number of factors that are brought about by rapid urbanization will increase number of the urban risks [3,18], while high-quality and intense urbanization will play an essential role in strengthening the level of urban resilience [49]. Instead, urban resilience will influence urbanization as well. A high level of resilience can create an environment for the safe operation of cities, thereby effectively improving the level of urbanization; while cities with a low level of resilience often have a higher probability of risk occurrence and have poor risk prevention and scientific management and control capabilities, which limits the improvement of the level of urbanization.

From the standpoint of the subsystems, the urbanization subsystem and the urban resilience subsystem have a complicated interaction. In one sense, urbanization affects urban resilience at multiple levels of the economy, population, space and society. Economic urbanization brings great economic benefits and development opportunities to the cities, thereby enabling cities to have sufficient funds to support industrial restructuring, urban planning and infrastructure construction [50], which can help the city to improve the economy, infrastructure and social resilience to a certain extent. However, the county’s single industrial structure and extensive resource utilization causes environmental pollution [51], which in turn leads to a reduction in the level of ecological resilience. Population urbanization results in the movement and concentration of a significant number of people and resources in the urban regions, thereby promoting economic and social resilience. At the same time, the increase in the population density will inevitably put pressure on the environment and urban public facilities [18] and reduce the ecological and infrastructure resilience. Spatial urbanization, as the spatial representation of urbanization, improves the level of urban infrastructure resilience, but unscientific urban planning and uncontrolled urban sprawl will increase the degree of ecological pressure that is on the city [52], thereby resulting in an insufficient level of urban ecological resilience. Social urbanization influences the people’s values, including their ecological beliefs, and this contributes to the improvement of the urban ecology and social resilience.

**Figure 1:**
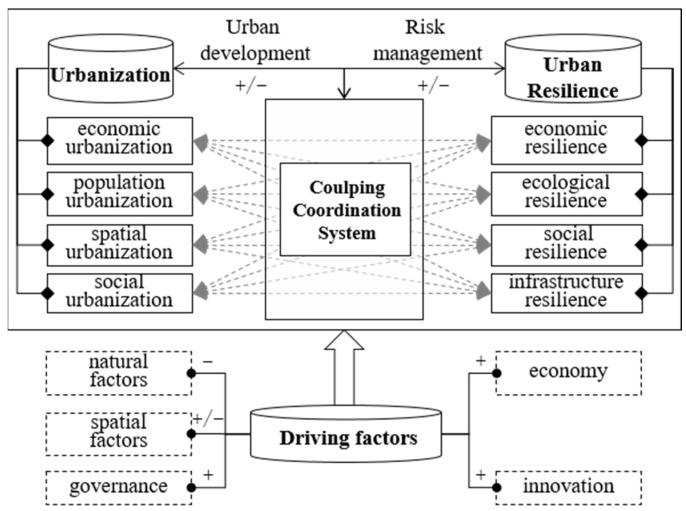
The relationship framework between urbanization and urban resilience. “+” means promote, “−” means restrain, while “+/−” means promote or restrain.

Urban resilience, in turn, has a corresponding response to the effects of urbanization. Economic resilience promotes or constrains the level of economic urbanization by improving the economic structure, and then, it rationally allocates the distribution of the population in spaces. Ecological resilience helps to eliminate the negative impacts of ecological degradation and contamination as a result of urbanization, and rationally allocates the urban ecological spaces by responding to the occurrence of malignant environmental events [53]. Infrastructure resilience guides the scale and layout of the urban spatial urbanization by responding to the urban risks such as waterlogging disasters and meteorological disasters. Social resilience constrains the degree of the consumption intensity of the human activities on nature by guiding the transformation of the urban populations’ values. The relationship is shown in Figure 1. In addition, the interaction that is between the urbanization and urban resilience subsystems is dynamic. For example, in the early stage of economic urbanization, a low-level industrial structure has an impact on the environment, with there being an advanced transformation of the industrial structure, and economic urbanization has a positive impact on the level of ecological resilience. Therefore, it is crucial to study the coupling relationship from the standpoint of the spatial-temporal evolution.

Based on the literature review and the theoretical framework, we put forward theoretical assumptions: (1) The coupling coordination degree between urbanization and urban resilience is closely related to the development stage of the county, and the overall level of it in Hebei Province is not high, which means that it is accompanied by spatial differences. (2) The natural elements, the spatial forms, government governance, the economic level and the innovation ability of the area are the core elements affecting the coupling coordination degree, among which the natural elements have a negative impact, the nature of the governance, the economic level and the innovation ability have a positive impact on it, while the urban form has an uncertain impact on the coupling coordination degree.

## 3. Materials and Methods

### 3.1. Research Area

China is currently promoting a county-based urbanization, however, many problems urgently need to be solved at the county level, but the existing research has paid little attention to this important scale, thus it is crucial to study the coupling between urbanization and urban resilience from a county perspective. Hebei Province has 167 counties which are under the jurisdiction of 11 cities, which are all located in the eastern part of China. The urbanization character of the counties in Hebei Province have experienced a transition from them having an extensive development to a sustainable one. In the early stage of urbanization, Hebei Province was in a weak position as it was siphoning the BTH’s urban agglomeration because of the relocation of its polluting enterprises from Beijing and Tianjin [51,54], and the government’s low governance level and weak infrastructure also made it less capable of coping with the risks that are associated with this [51,55]. After 2014, along with the regional coordination strategy of BTH, the urbanization strategies in Hebei Province turned towards the goal of achieving sustainable development. In the 2020 national land and space planning framework, it was positioned as a support area for the Beijing–Tianjin–Hebei ecological environment. Both the territorial space planning of Hebei Province and the new urbanization of the county-level entities have put forward the requirement of “building a safe and resilient city”. Therefore, it is important to assess how the county-level urbanization and urban resilience are coordinated in a scientific way, which is essential for the BTH region’s sustained growth. In addition, given that the county area of Hebei Province is a typical area of rapid urbanization development in China, the research results that are based on this area can also provide a reference for the sustainable urbanization of other developing cities in the world.

**Figure 2:**
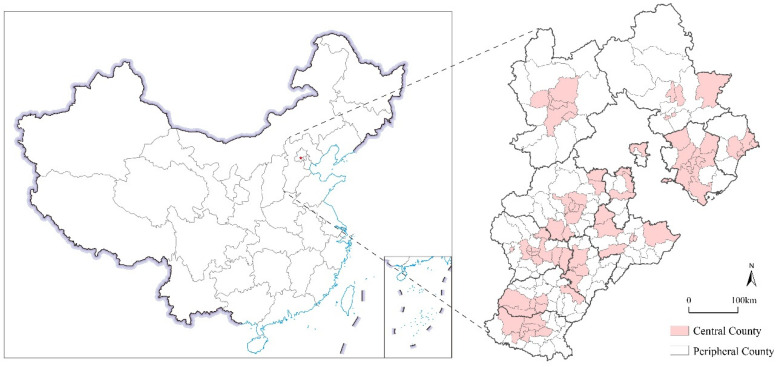
Study area.

Due to there being different development levels, for the convenience of comparison, this paper lists the municipal districts and county-level cities of 11 prefecture-level cities as central counties (70), and the rest of the counties as peripheral counties (97). Figure 2 visually shows the study area.

### 3.2. Construction of Evaluation Indicators

As the previous literature review suggests, the mainstream measurement system for urbanization and urban resilience varies under a unified framework. Specifically, the unified framework for urbanization is an assessment of the city’s economy, population, space and other areas, while that of the factor of urban resilience focuses on the degree of resistance, coping, recoverability and adaptability of the urban economic, social, natural and other systems when they are facing disasters [56], and the difference is reflected in the selection of each subcategory indicator. Therefore, we summarize the classic literature in this field and the metrics that they adopt. For urbanization, the covered indicators are summarized by referring to the classical index [57,58,59] which has been developed previously; Table 1 displays them in detail. For urban resilience, the currently accepted mainstream measurement frameworks include the Rockefeller resilient city framework, DROP (local resilience) [60], BRIC (baseline resilience indicator of community) [61] and the improved local resilience framework [62,63]. The indicators that are covered by these studies are as shown in Table 2.

Based on the data availability and representation, the county-level urbanization evaluation system was built with four factors in mind: (1) The new type of urbanization has significantly boosted the economic growth. According to a previous study [65], the GDP (A1) and the proportion of secondary and tertiary industries in the GDP (A2) were used as assessment metrics for economic urbanization. (2) Population growth is the core of urbanization, which was expressed by the proportion of the urban populations (A3) [3]. In addition, we took the ratio of the employed population in the secondary and the tertiary industries in the total population (A4) as the representation of the industrial population urbanization [66]. (3) In terms of spatial urbanization, the degree of urbanization is closely related to the land use, therefore, the urban area of the construction land (A5) and the urban construction land per capita (A6) were used to represent the level of spatial urbanization [13,67]. (4) In terms of social urbanization, the per capita retail sales of the consumer goods (A7) and the social welfare units (A8) were adopted to measure the level of social urbanization [65,68].

We integrated the existing research on institutional resilience and social resilience into the social resilience dimension in the study, and the county-level urban resilience evaluation system was constructed from four dimensions: (1) Economic resilience mainly represents the city’s economic stabilization when it is facing the impact of uncertain economic factors. This can be divided into four parts including the economic development level, the growth level, the recovery capability and the industrial production capacity which can be assessed by the per capita GDP (B1) [69], the public financial revenue (B2) [27], the household savings (B3) [28], and the industrial output value that is above a designated size (B4) [49]. (2) The ecological risks in the urban development process are mainly expressed in the destruction of the ecosystem which is caused by urban expansion and the discharge of pollutants which are caused by industrial production. Therefore, four indicators were selected to evaluate the level of ecological resilience, namely, NDVI (B5), which reflects the pre-disaster mitigation capacity of the natural ecosystem [70], PM_2.5_ (B6) and carbon emission (B7), which measure the level of green industrial development [53,71] and the green coverage rate of the urban construction area (B8), which reflects the disaster reduction capacity of the urban ecosystem [27]. (3) Social resilience is mainly reflected in the social potential and recovery capability of the cities when they are facing disasters. Therefore, based on the availability of the county data, the total population value (B9) was selected as the city’s degree of vulnerability to disasters [27], the number of students in middle schools per 10,000 people (B10) was selected as the city’s pre-disaster preparedness capacity [33], and the number of beds in medical and health institutions per 10,000 people (B11) was selected as the city’s disaster response capacity [27]. (4) The degree of infrastructure resilience is mainly manifested in the capacity to offer infrastructure support when disasters occur. Three indicators were selected to evaluate it, including night light (B12), which presents the disaster-carrying capacity of the urban space [72], the road mileage per capita (B13), which is the post-disaster response capability of the infrastructure [70], and Internet access rate (B14) [61]. Table 3 displays the assessment indicators in detail.

### 3.3. Driving Factors

As reported in Table 4, in the selection of the driving factors, six explanatory variables were selected from five levels, which are nature, space, government, economy and innovation. In terms of the natural factors, we selected the roughness of land surface, which is expressed as “RDLS” [40]. In terms of the spatial factors, both the urban structure and the urban form have an impact on the coupling relationships, while the urban structure is difficult to quantify in comparison, thus, we selected the quantitative spatial indicators of the urban form with mature standardization methods. The “area-weighted mean shape index (AWMSI)” was selected to represent the complexity of the urban form. The “compactness index (BCI)” was selected to represent the compactness of the urban form. The two indicators have been shown in previous studies to be typical indicators of the urban formation [73] and both indicators were calculated using the FRAGSTATS software. We selected “government public financial expenditure (ZC)” to represent the governance capacity as it is the most important embodiment of the governance ability. When it comes to the economic variable, previous studies have measured the advanced degree of the urban industrial structure from multiple indicators [74,75]; “the ratio of output value of the tertiary industry to the secondary industry (CY)” is the most important index among them. In terms of innovation, previous research has measured the ability of scientific and technological innovation by the proportion of scientific investment in the governments’ expenditure and the number of patents [76], while these data are largely missing at the county level, thus, it was replaced by “the number of new scientific and technological innovation enterprises (CX) in the current year” in this paper.

### 3.4. Data Sources

The socio-economic statistics were mostly collected from the 2010–2020 China Counties and Hebei Province Statistical Yearbook as well as prefecture-level city statistical yearbooks. Some missing data (green coverage in urban areas) were mainly calculated through the use of mathematical operations which were applied to the indicators. These were calculated by applying the processes of superposition and synthesis, and the individual missing values were filled by a linear interpolation; the county scale PM_2.5_ data came from the Atmospheric Composition Analysis Group (http://fizz.phys.dal.ca/~atmos/martin/ (accessed on 15 May 2022)); the carbon emission data came from the open-source data which were developed and calculated by Chen. et al., which were based on the PSO-BP model [77], the data’s accuracy is also at the county scale; the night light data came from the NPP-VIIRS fusion image which was developed by Chen. et.al [78], and the grid resolution of this is 1 km; the NDVI and land use cover data for calculating urban form came from the Resource and Environmental Science and Data Center of the Chinese Academy of Sciences (https://www.resdc.cn/ (accessed on 17 May 2022)), and the grid resolutions of this are 1 km and 30 m, respectively. Specially, the NDVI is the annual average due to there being missing data, e.g., the NDVI data in 2020 was replaced by the data in 2019. The innovation level was represented by the number of newly added enterprises in the wind database.

### 3.5. Methods

#### 3.5.1. Entropy Weighting Method

To reduce the effects of the subjective variables, we used the entropy method to estimate the weight of them, which was determined using the information entropy value. This is an objective assessment of the source of the information’s uncertainty [79], which can assess the degree of change and information entropy to calculate the indicator weight [80]. A min–max normalization adjustment was applied to the original data to eliminate statistical error that is caused by the differing calculation index units.

#### 3.5.2. Coupling Coordination Degree Model (CCDM)

“Coupling”, which is a concept that is derived from physics, describes the situation when numerous systems interact with one another. The coupling coordination model, which is also called CCDM, can be used to measure the coupling coordination degree (CCD) to reflect the interaction level between two systems [11]. In this paper, this model was used to comprehensively calculate the CCD between urbanization and urban resilience. The specific model is as follows:

(1) $$C=\sqrt{\frac{U1U2}{{\left[\left(U1+U2\;\right)/2\right]}^{2}}}$$

In the formula: *U*1 and *U*2, respectively, represent the level of urbanization and urban resilience, which were calculated by the entropy method. *C* denotes the degree of coupling, which spans from 0 to 1. The *C* value indicates the strength of the connection between the two subsystems.

We utilized the CCDM to further investigate this since the *C* value can only show the existence or the lack of a connection across the systems, but it cannot measure the degree of it. The formula for calculation is as follows:

(2) $$D=\sqrt{\mathrm{CT}}=\sqrt{C(\alpha U1+\beta U2)}$$

where *T* is the total assessment indicator of urbanization and urban resilience, while *D* denotes the degree of coupling coordination. *α* and *β* are their weights in the comprehensive evaluation, which both take 1/2 of it. The evaluation criteria of the CCD can be separated into six stages as shown below [81] (Table 5).

#### 3.5.3. Spatial Autocorrelation Analysis

Determining the geographical distribution and the relationship within this is crucial for spatial data, which can be assessed by a spatial autocorrelation. In this article, the global spatial autocorrelation was used to describe the geographical characteristics of the attribute values in Hebei Province so as to test the correlation between the attribute values of the spatial units and the adjacent units [82] using *Moran’s I* value as the calculation index to calculate the global spatial autocorrelation. It is determined as follows:

(3) $$\mathrm{Moran}’s\;I\;=\;\frac{\sum_{i=1}^{n}\sum_{j=1}^{n}W_{\mathrm{ij}}\left(X_{i}-\bar{X}\right)\left(X_{j}-\bar{X}\right)}{S^{2}\sum_{i=1}^{n}\sum_{j=1}^{n}W_{\mathrm{ij}}}$$

*n* represents the amount of county study units; *X_i_* and *X_j_* represent the coordination degrees of *i* and *j*, respectively; *W_ij_* denotes the spatial weight matrix; *S^2^* is the variance of the coordination degrees; $\bar{X}$ is the average of the coordination degrees. When *Moran’s I* > 0, it is positively correlated, thus the greater the value is and the stronger the geographical association is; while the contrary it also true, the correlation is negative when the value is smaller, and thus, the bigger the geographical difference is; whereas when *Moran’s I* = 0, it distributes randomly.

The global *Moran’s I* reflects the existence of the autocorrelation features in the global space, and when both of the positive and negative correlations exist in the global attribute data, these two may weaken the effect of each other, thus making the global *Moran’s I* inaccurate. To handle the problem, Anselin proposed the local *Moran’s I* [83] to observe the relationship between the unstable features of local space. This index can represent the intrinsic properties of the global autocorrelation and discover the spatial correlation patterns in various global local regions. The calculation process is shown in the formula:

(4) $$I_{i}=\frac{\left(X_{i}-\bar{X}\right)\sum_{j=1}^{n}\left(X_{j}-\bar{X}\right)}{S^{2}}$$

The variables in the above-mentioned Formula (4) have the same meaning as they do in the previous Formula (3). *I_i_* > 0 represents the spatial clustering of the positive correlations of the adjacent cells in the local region; *I_i_* < 0 represents the spatial clustering of the negative correlations of the adjacent cells in the local region; *I_i_* = 0 represents the spatial clustering of the values of the adjacent cells in the local region random distribution.

#### 3.5.4. Spatial Durbin Model

The presence of the spatial factors may impair the accuracy of the estimated results of the general panel regression models. Therefore, we constructed a spatial panel regression model for the analysis of them which can better test the relationship between the variables and the spatial effects. For the spatial weight matrix, the geographic adjacency matrix (W1) and inverse distance matrix (W2) were generated using the latitude and longitude data from 167 counties in Hebei Province. After the LM test and LR test were conducted, we finally selected the spatial Durbin model (SDM). The spatial Durbin model considers both the influence of the dependent variable’s and the independent variable’s spatial lag terms on the dependent variable, and it is commonly used to examine the independent variable’s geographical spillover impact [84]. Further through the Hausman test, the model that was finally determined in this paper is the space Durbin model of the individual time double-fixed effect. The model is calculated as follows:

(5) $$\begin{array}{c} Y_{\mathrm{it}}={\;\;\;\beta \;\;\;}_{0}+\;\;\;\rho \;\;\;\sum_{j=1}^{n}\;W_{\mathrm{ij}}Y_{\mathrm{jt}}+\;\;\;\theta \;\;\;X_{\mathrm{it}}+\;\;\;\varphi \;\;\;\sum_{j=1}^{n}\;W_{\mathrm{jt}}X_{\mathrm{jt}}+{\;\;\;\alpha \;\;\;}_{i}+{\;\;\;\delta \;\;\;}_{t}+{\;\;\;\varepsilon \;\;\;}_{\mathrm{it}}\\ \;\;\;\;\;\;\; \end{array}$$

Among them, the counties are represented by *i* and *j*, *W_ij_* denotes the matrix, *Y_it_* represents the explained variable, which is the CCD between urbanization and urban resilience, and *X_it_* is each explaining variable. *θ* is the explaining variable’s regression coefficient without considering the spatial effect, *ρ* and *φ* are the geographic regression coefficients of the explained variable and explanatory variable, which are the dependent and independent variable’s space spillover impact, respectively. *α_i_* represents the county individual fixed effect, while *δ_t_* stands for the time fixed effect, and *ε_it_* is the random disturbance term.

## 4. Results

### 4.1. Overview of Urbanization and Urban Resilience

#### 4.1.1. Urbanization’s Spatial-Temporal Development

**Figure 3:**
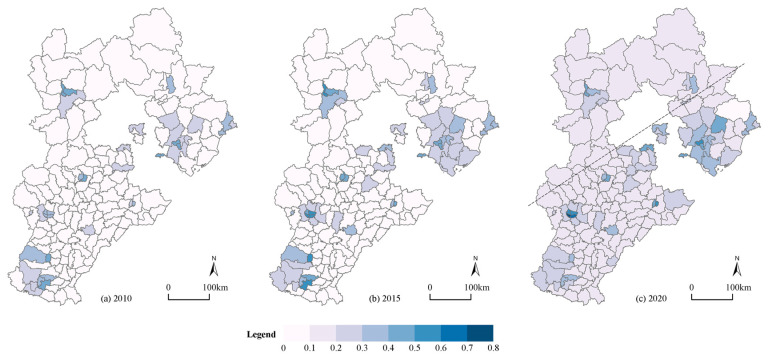
Spatial distribution of the urbanization of 167 counties in Hebei. (**a**) Urbanization in 2010; (**b**) urbanization in 2015; (**c**) urbanization in 2020.

In terms of time, the average urbanization level of the 167 counties in Hebei Province increased from 0.15 in 2010 to 0.21 in 2020, thereby demonstrating that although most counties’ urbanization levels have improved, their aggregate levels are still low. The urbanization level’s standard deviation rose from 0.097 to 0.10, thereby indicating that the urbanization level difference between the counties was not large, but the gap between them further increased during the study period. At the spatial level, it generally presented a state of it being “lower from southeast to northwest” with “Pingquan City-Pingshan County” acting as the dividing line between these regions. The urbanization level of the central counties was always higher than those of the peripheral counties, and the trickle-down effect of the central counties was obvious, which formed a spatial pattern that had a “center-periphery” pattern. This case is depicted in Figure 3. In general, the urbanization levels of the counties in Hebei Province were not high, and they showed a significant feature that was localized in the space of the central county which drove the urbanization that occurred in the peripheral counties, and there was still a lot of opportunity for improvement during the course of the new-type urbanization with certain counties being a significant carrier.

#### 4.1.2. Urban Resilience’s Spatial-Temporal Development

**Figure 4:**
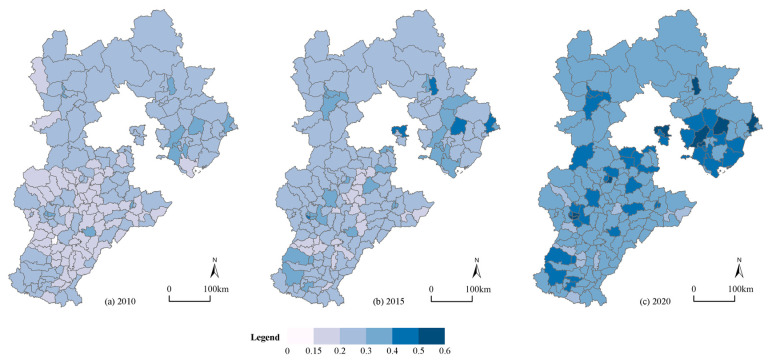
Spatial distribution of the urban resilience of 167 counties in Hebei. (**a**) Urban resilience in 2010; (**b**) urban resilience in 2015; (**c**) urban resilience in 2020.

In terms of time, the average urban resilience level of the 167 counties in Hebei Province increased from 0.22 in 2010 to 0.38 in 2020, thereby indicating that the degree of resilience in most of the counties improved, while the aggregate level was still low. The standard deviation of the level of urban resilience rose from 0.043 to 0.065, thereby demonstrating that the gap in urban resilience between the counties tended to increase. At the spatial level (Figure 4), the spatial distribution of the urban resilience factor differs from that of the urbanization one, showing a state whereby the “south is low while the north is high”, which partly shows that the urbanization in this region is not synchronized with the urban resilience development. Counties that are located in the middle of Hebei Province like Gaoyang County have achieved a leap from a lower degree of resilience to a higher degree of it, while some of the southern counties like QuZhou County still had a low level of resilience in 2020. The resilience level of the central counties has always been higher than that of the peripheral counties, basically that which is above 0.5, while there was a higher level than the average of 0.34 in the peripheral counties, thus showing a “center-periphery” distribution feature locally. Qinhuangdao City, which is in the northern part of Hebei Province, is an ecological model city, while the central part such as Baoding City and Shijiazhuang City have actively promoted the industrial upgrading framework, thereby moving from a low-quality urbanization to a green, sustainable and innovative urbanization development path, which promoted the level of resilience. In conclusion, the average degree of the urban resilience in Hebei Province counties was not high, showing a strong spatial differentiation in space, and it was not completely consistent with the geographical differentiation of urbanization. The level of urban resilience was related to the development stage of each county and its specific conditions.

### 4.2. The Spatial-Temporal Evolution of Coupling Coordination Degree

#### 4.2.1. Coupling Coordination Degree

From 2010 to 2020, the coupling of urbanization and urban resilience in 167 counties in Hebei Province has grown steadily. The average coordination degree has risen from 0.415 to 0.520, and the overall level of this manifested as a mild imbalance. The type of coupling coordination has improved in each county, which evolved from there being a moderate imbalance in the main body of it in 2010 to there being a mild imbalance in the main body of it in 2020, and the counties with a mild imbalance, a mild degree of coordination and a moderate degree of coordination have demonstrated a significant increase in these. The central counties increased from 0.473 to 0.578, while the peripheral counties have developed from 0.374 to 0.479. The average degree of coordination in the central counties was greater than that in the peripheral counties, but the growth rates of the two were the same during the course of the research. This showed that there were significant differences in the coordination status among the counties, which were closely related to the differences in the city size and their economic development level. This verified our hypothesis on coupling coordination.

**Figure 5:**
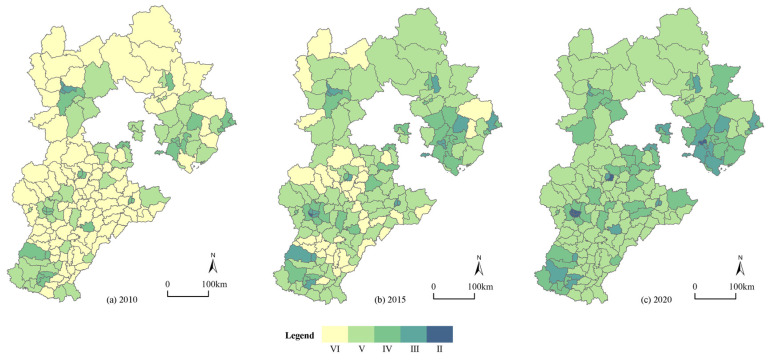
Spatial distribution of the coupling coordination degree between urbanization and urban resilience of 167 counties in Hebei. (**a**) CCD in 2010; (**b**) CCD in 2015; (**c**) CCD in 2020.

From a spatial standpoint, there were significant spatial-temporal disparities which existed in the coordination of urbanization and urban resilience in Hebei Province, showing the distribution characteristics of it being “high in the east and low in the west, high in the middle and low on the outskirts” (Figure 5). In 2010, the coordination level of the whole province was low, all the peripheral counties were serious imbalanced, and only a few central counties such as Qiaodong District achieved a mild coordination, which indicated that at this time, the central county had a first-mover advantage due to the expenditure of the policy funds in this area. In 2015, most of the county towns achieved a leap from a serious imbalance to a mild imbalance, which may be the result of the central counties encouraging the resources to radiate to the peripheral counties, while the centrally located counties such as Yi County and Dingxing County were still in a state of serious imbalance. In 2020, all of the 167 counties were above the level of a mild imbalance. With “Pingquan City-Pingshan County” acting as the boundary, the difference in the degree of coordination between the east and the west was further expanded. Additionally, the disparity between the central counties and the peripheral counties has grown, which made the central–peripheral structure more pronounced. This may indicate that while the trickle-down effect exists, some central counties also have a siphon effect.

#### 4.2.2. Coupling Coordination Type Evolution

In order to observe the evolution of the coupling coordination types, we divided them further. In 2010, the VI-1 and VI-3 coordination types accounted for the largest proportion of them, which was followed by type V-1, and the whole province was in the stage of a serious imbalance between urbanization and resilience. In 2015, the V-1 coordination type accounted for the largest proportion of them, and the whole province was in the stage of a moderate imbalance of urbanization and resilience. In 2020, the V-3 coordination type accounted for the largest proportion of them, and coordination level improved in the whole province, but the urbanization factor was lagging behind.

**Figure 6:**
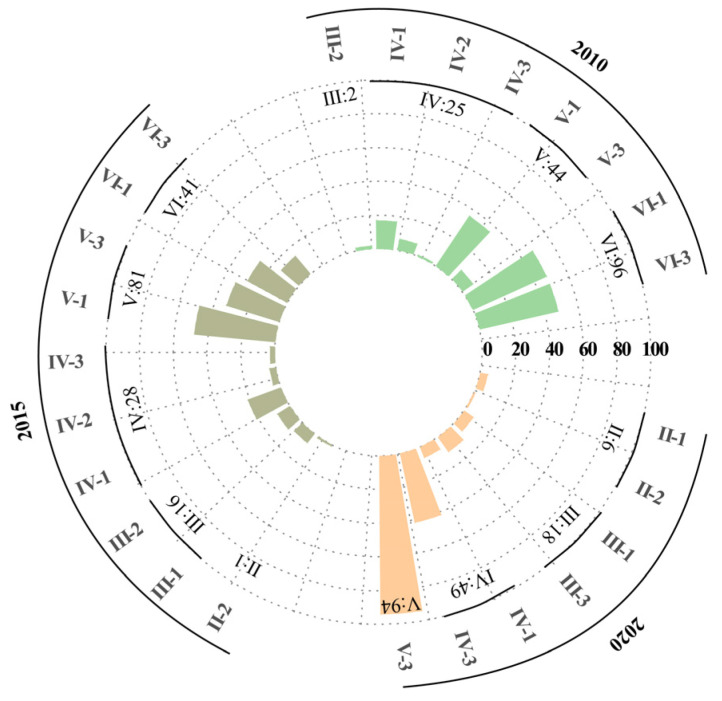
Coupling coordination degree type in 2010, 2015 and 2020.

**Figure 7:**
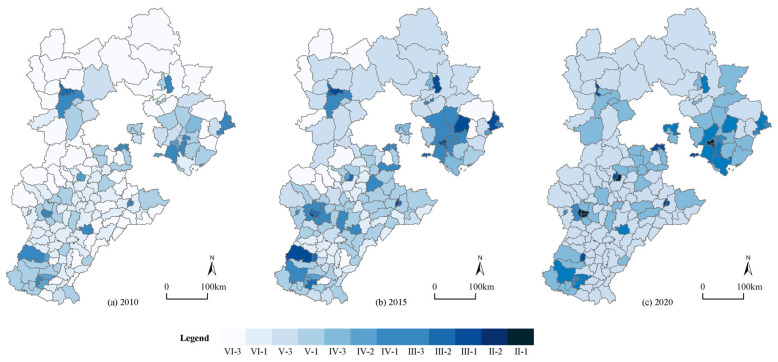
Spatial distribution of the coupling coordination type of 167 counties in Hebei. (**a**) Coupling coordination type in 2010; (**b**) coupling coordination type in 2015; (**c**) coupling coordination type in 2020.

From the spatial point of view, in 2010, due to the disasters which are derived from the process of rapid urbanization, the resilience levels of the central counties were lagging behind, while the northwestern peripheral counties such as Kangbao County were lagging behind in their urbanization level due to their better natural background conditions. Neither the central counties nor the peripheral counties can take into account the balanced development of urbanization and urban resilience at that time. In 2015, each county showed a state of diversity. The urbanization and resilience of the central counties were greatly improved, but the level of resilience was still lagging behind the former; some peripheral counties, such as Wen’an County, were in the state of balanced urbanization and resilience, which benefitted from the overflow of the industrial population in the central county, along with the simultaneously improvement of the ability of the urban governance and disaster response due to the rapid development of urbanization; while in other peripheral counties such as Yangyuan County, the urbanization factor still lagged behind the urban resilience one. In 2020, the central counties completed their transformation to high-quality urbanization, and achieved the balanced development of urbanization and urban resilience, which made five counties, including Qiaodong District, have achieved a mild coordination; while for most of the peripheral counties, although the coupling coordination degree improved from a serious imbalance to an all-mild imbalance, the degree of urbanization was still lagging behind the degree of urban resilience due to the lack of a corresponding development basis and policy support. Figure 6 and Figure 7 visually represent this evolution.

#### 4.2.3. Spatial Autocorrelation Analysis of CCD

Table 6 shows that the *Moran’s I* values which are based on the adjacency spatial weights matrix were all significantly greater than 0 between 2010 and 2020. This indicates that the distribution of the CCD between county-level urbanization and urban resilience in Hebei Province is positively correlated, with there being strong spatial aggregation characteristics, and the trend intensified year after year.

**Figure 8:**
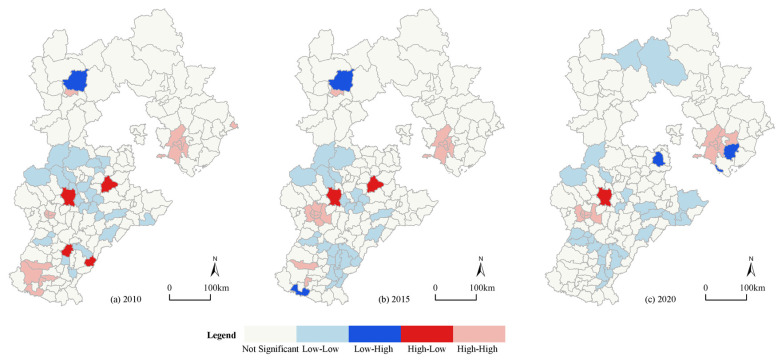
Spatial distribution of the coupling coordination degree of 167 counties in Hebei. (**a**) Spatial distribution in 2010; (**b**) spatial distribution in 2015; (**c**) spatial distribution in 2020.

The local Moran’s I showed that the distributions of the H-H clusters and the L-L clusters were basically the same in 2010, 2015 and 2020, and the dominance of the L-L cluster did not change (Figure 8). This indicates that although the overall coupling coordination improved, a negative overflow still exists in some areas. The H-H clusters were mainly distributed in Shijiazhuang, the Tangshan municipal districts and the surrounding counties. Most of these counties are central counties with better policies and more financial support for urban development, which are coupled with good resource conditions, hence, the levels of urbanization and urban resilience have always maintained a high balance. The L-L clusters were distributed in the middle and western regions like Fuping County and Boye County. In comparison with the central county and the other peripheral counties, they have location disadvantages, poor resource conditions and a lack of urbanization policy support, which has led to the formation of the L-L agglomeration areas. The number of L-H and H-L clusters decreased during 2010–2020, thereby showing the regional coordination trend of the CCD.

### 4.3. Driving Force of Coupling Coordination Degree

After verifying the degree of the spatial correlation using the spatial autocorrelation analysis, we developed a spatial panel model to investigate the CCD-driving mechanism. In order to verify the result, we used two spatial matrices in the model, including the adjacency spatial weight matrix (W1) and inverse distance weight matrix (W2).

As shown in Table 7, according to the results of the LM test, the LR test and the Hausman test, all of the statistics achieve a 1% significance threshold, demonstrating that the fixed SDM should be employed for the driving factor analysis. The findings of the SDM model which was based on the W1 matrix are shown in Table 8. For comparison, the time fixed effect, the individual fixed effect and the double-fixed effect were listed.

The spatial autocorrelation coefficient of the CCD between urbanization and urban resilience at the county level in Hebei Province is 0.157, thereby confirming that it has a considerable regional spillover impact. For every 1% increase in the CCD between local urbanization and urban resilience, the degree of coordination in the neighboring counties will increase by 0.157% on average.

In terms of the main effects of each variable, the amount of government financial expenditure, the industrial sophistication level and the innovation level all have a substantial beneficial influence on the CCD, while urban compactness has a significant negative impact on it, which is basically consistent with the previous theoretical hypothesis. According to the 100 Resilient Cities (100RC) Programme by the Rockefeller Foundation [85], the government promotes industrial specialization through public financial expenditure, and it also carries out urban planning and infrastructure planning to enhance its ability to deal with urban risks, thus enhancing the CCD. The advancement of the industrial structure is a symptom of the development of a high-level urbanization, and on the one hand, it improves the utilization efficiency of the resources [86] and reduces the output of pollution [87], and on the other hand, it promotes poverty reduction in the urban population [88], which can enhance the degree of urban economic and social resilience. Innovation is one of the key drivers of urban growth [89]; it exists widely at the technical and social levels, and technological innovation is closely linked to the economic value, and social innovation improves people’s perceptions of cities and their attraction to them [65]. In both respects, innovative behavior will significantly improve the CCD of urbanization and urban resilience. The impact path of the urban form is complex, and it generally presents a negative impact, that is, the more compact the urban form is, then the lower the CCD of urbanization and urban resilience is.

From a perspective of the spillover effects of various variables, government financial expenditure has a substantial positive impact on the CCD of the neighboring counties. A county’s government can improve the coupling coordination degree of the adjacent counties by guiding the transformation of new industrialization practices and the construction of regional infrastructure through the allocation of public finance. This provides a path for the improvement of the regional areas of urbanization and the resilience coupling coordination.

Finally, we used the inverse distance weight matrix to test the re-regression and robustness of the data. The coefficients of the regression results were basically consistent with the adjacent weight matrix, and the regression passed the robustness test (Table 9).

## 5. Discussion

The relationship between urbanization and urban resilience is an emerging field, and it is also crucial during the practicing of new-type urbanization to consider counties as the significant carriers of it. As the peripheral region of the BTH, the counties of Hebei Province are an important component of the implementation of the county urbanization development strategy at this stage. The coordination of urbanization and resilience can ensure urban security. Therefore, it is necessary to investigate the development stage and the driving factors of the coupling between urbanization and urban resilience in different periods and regions of Hebei Province.

### 5.1. Discussion on Evaluation Index System

In terms of the index evaluation system, this paper has based it on the provincial county units, and it innovatively integrates urbanization and urban resilience together, thereby comprehensively evaluating the two categories from a macro perspective. Given that there is currently no standard on how to quantify urban resilience in academia, we adopted a mainstream classification of the subsystems including economy, ecology, society and infrastructure. However, as we reviewed in Section 3.2, urban resilience is a complex framework that covers multiple levels of indicators, and different studies have demonstrated the influencing factors of urban resilience from various perspectives, thus the selection of indicators has a certain degree of influence on the results and it needs to be further discussed.

Based on the availability of the data, the reproducibility of the previous studies, and the redundancy of the indicators, we selected the core metrics. It should be noted that while many indicators are available at the city or provincial level, this is not the case at the county level. The lack of relevant information to provide indicators is one of the biggest difficulties we faced in the research that we conducted, and it has also been a major challenge in previous county-level studies [90]. The indicators in the BRIC are mostly available through open-source data [61], however, China has less access to open-source indicators in this regard. Secondly, we selected the indicators that were frequently involved in the previous studies. If an indicator was adopted in multiple studies, this indicates that it is meaningful in various geographical environments. Finally, we took full account of the redundancy of the indicator itself, choosing one or two necessary indicators in each subclass. For example, we took PM_2.5_ instead of both PM_2.5_ and PM_10_ as the representative of the level of green industrial development, and this is because of the similarity of the two metrics, and we do not think that it has affected the accuracy of the results. Nonetheless, our indicator system proves that it can be widely applied to county-level research in China.

### 5.2. Interaction between Urbanization and Urban Resilience

The temporal evolution of the CCD shows that it basically presents a phased trend, which is related to China’s five-year socio-economic development plan. The spatial evolution demonstrates a pattern of a spread that has moved from east to west and from the central to the edge areas, thereby indicating that the coupling coordination degree has an obvious “trickle-down” effect at the regional level, which is consistent with previous studies [50]. Driven by the central county, the coupling coordination degree in Hebei Province has crossing from a serious imbalance to a mild imbalance. The evolution of the interaction between urbanization and urban resilience can be inferred from the type of coupling coordination, which is corroborated by Ren et al. [91], Mu et al. [92] and others from the same period.

At the beginning of the study in 2010, Hebei province was in a period of accelerated urbanization and a low level of urban resilience. The central county had a first-mover advantage and it had a high urbanization rate in the early stage of the research. However, at the same time, the central county was in the period of rapid urbanization, and its governance ability to deal with environmental pollution and disaster risks was weak, thus the resilience of the city lagged behind in terms of urbanization. While the peripheral counties showed a contrary pattern, whereby the ecological conditions of them were relatively good then, and the urbanization level was relatively low without there being the pressure of a massive population and resource agglomeration, thus the urbanization and urban resilience strategies were developed in a relatively coordinated way.

At the middle of the study in 2015, Hebei Province was in a period wherein the urbanization risks emerged, and the level of urban resilience improved. In the year of 2014, with the advancement of the regional coordination of the BTH and the need for environmental governance in Beijing and Tianjin, numerous polluting low-end businesses were transferred to Hebei Province [51]. When the environmental resources which were consumed by industrial development exceeded the expectations, the ecological degradation and disaster risks increased. Some counties focused on pollution control and risk prevention, and they gradually achieved a relative balance between urban resilience and urbanization, while other counties ignored the risk management, thereby resulting in a lagging level of resilience. Due to the far distance with the central county, some peripheral counties did not receive an overflow of resources, and they were even effected by the “siphonage” effect, which made the population and resources become further concentrated in the central county, hence the level of urbanization lagged behind that of urban resilience.

At the end of the study in 2020, Hebei Province was in the period wherein the level of urbanization was lagging behind that of urban resilience. In 2016, the People’s Republic of China’s State Council proposed ideas for new types of urbanization to accelerate the county’s infrastructure development, which drove the counties’ urbanization strategies to transform from those that were characterized by speed to those that were characterized by quality, and this also resulted in the improvement of the resilience and risk management abilities of the counties’ infrastructure. At the same time, after the 19th National Congress of the CPC, the construction of an ecological civilization was achieved, and air pollution levels had been reduced [3], along with the improvement of the degree of ecological resilience, which made the central counties’ level of urban resilience become gradually developed in coordination with the urbanization practices. However, for most of the peripheral counties in the southwest and northwest, such as Xinhe County, the location disadvantages and the lack of urbanization policy support led to there being a slow urbanization process, and the urbanization level relatively lagging behind those of other areas.

### 5.3. Discussion on Driving Factors

Overall, the governance, the economic development, and the innovation levels have a positive effect on the CCD, which is consistent with the Rockefeller Resilience Framework [23] and the resilience cities review [24]. While the mechanism of the urban form on the CCD is somewhat complex and it has been little involved in previous studies, thus additional discussion is required.

The sprawl or agglomeration of the urban form has a profound influence on the CCD. The AWMSI has a considerable favorable effect on coordination, while the BCI has a strong detrimental influence on it. It has been shown that a compact urban form improves land use efficiency and leads to sustainable urban development [93]. However, studies have shown that an increased urban density may degrade the quality of the open and green places, thereby leading to the reduction in temporary shelters after a disaster occurs, thus reducing the city’s capacity to mitigate the disaster risks [94]. Relatively speaking, the resilience level of the scattered urban forms is higher, especially with the placement of more green spaces in urban open spaces [95]. Meanwhile, the scattered urban form can reduce the generation of PM_2.5_ pollution in small cities as well [44]. Therefore, the influence path of the urban form on the CCD can be roughly determined, the advancement of urbanization has led to the agglomeration of resource elements, and urban forms tend to develop compactly, spontaneously or under the guidance of the government, which may mean that the public evacuation land is squeezed, therefore, the city’s resistance and adaptability to disasters is reduced, and this also reduces the level of ecological resilience. This multifaceted superposition has led to a reduction in the level of urban resilience, and in turn, this leads to the incompatibility between urbanization and resilience development.

It should be pointed out that the study of the urban form and resilience is a field that few people have been involved in, and the only research that has been conducted is limited to the impact of small-scale space on urban resilience, such as Gharai’s research on the multi-scale resilient urban space [96] and Sharifi’s literature review on the street form and the level of urban resilience [97]. The existing research on the relationship between the urban form and resilience at the regional scale is not sufficient. In future research, more indicators of the urban form and the land use pattern can be developed by focusing on the urban spatial elements so as to deeply quantify their coupling relationship with urbanization and urban resilience.

### 5.4. Policy Implications

Based on the study’s findings, the following policy suggestions are offered:

(1) The government should multi-dimensionally promote the urban elements and coordinate the balanced development of urbanization and urban resilience. In terms of the spatial form, the government should optimize the land and space layout, delineate the urban development boundaries, and improve the urban form. In terms of governance, the government should increase its financial input to optimize the urban infrastructure structure, and improve the government water supply, gas supply and heat supply, as well as the urban functions such as flood control and temperature control, which are of great significance to improving urban resilience and enhancing the level of urban disaster resistance. The government should also focus on industrial upgrading and technological innovation as strong economic strength and a complete economic structure are important supporting forces for cities to resist risk shocks.
(2) Different policies are required by different counties, and the development strategies of them should be formulated based on each county’s own conditions. For the central county and its surrounding peripheral counties, which have good location advantages and industrial foundations, as well as a strong carrying capacity for the resources and environmental factors, it is essential to keep enhancing the quality and efficiency of the industrial supporting facilities and upgrade the municipal public facilities to ensure resilience in the process of urbanization. While the remaining peripheral counties, such as Zhangbei County in the northwest, should encourage the development of the ecological innovation belts, and enhance the urbanization process while guaranteeing urban resilience.
(3) The government should attach importance to the spatial spillover effect, and let the central counties drive the coordinated development of the peripheral counties. Each county should actively break the thinking constraints of the cooperation model that are brought about by the boundaries of the administrative jurisdiction, thereby maximizing the radiating and driving function of the central county. Relying on a more complete regional infrastructure network and a smoother flow of production factors will narrow the coordination gap between the counties by point and area and improve the network structure of a multi-party collaborative governance of urban resilience.

### 5.5. Lack of Research and Prospects

There are still some limitations in our study. First of all, in terms of the index evaluation system, on the account of the limitation by the availability of the county-level data and the diversity of the risks in different counties, the assessment system of urban resilience needs to be further improved, such as by adding evaluation indicators of the city’s capacity to deal with special risks. Meanwhile, this paper adopts a top-down indicator system to evaluate the urban resilience, but relevant studies show that a bottom-up residents’ organizational ability is also strongly connected to a city’s resilience [47], which is also one of the directions in which we could improve the indicator system in the future. Secondly, in terms of the research area, this study is based on the provincial county units, and we analyzed the spatial-temporal evolution and the spatial heterogeneity of the 167 counties’ CCDs from a regional synergy perspective, but this can be further focused to a study of a typical county that is aimed at performing the urbanization and urban resilience coupling coordination path analysis. Finally, in the exploration of the driving force mechanism, the selection of the driving factors needs to be further enriched. Meanwhile, other innovative methods such as the GTWR are needed to explore the spatio-temporal drivers of the CCD.

## 6. Conclusions

In this study, we assessed the level of urbanization and urban resilience at the county level in Hebei Province from 2010 to 2020. By combining the CCD and SDM models, the spatial-temporal evolution of coupling coordination between urbanization and urban resilience and its influencing mechanism were discussed, which has practical significance for promoting the construction of urban resilience and regional security improvement.

Firstly, the urbanization levels of the counties increased year after year, with the central counties’ levels always being greater than the peripheral counties’ were, therefore, they presented a geographical distribution that was “lower from southeast to northwest”. The level of urban resilience increased year after year, manifesting a geographical distribution that was “higher from south to north” and a local “core-edge” feature.

Secondly, the CCD increased steadily, and the degree of the central counties’ CCD was always higher than those of the peripheral counties. From the spatial view, it was bounded by the line of “Pingquan City-Pingshan County”, showing the distribution characteristics of it being “high in the east and low in the west, high in the center and low in the edge”. The evolution of the coupling coordination type has stages, and the level of urbanization is lagging behind, currently. The CCD has a positive spatial agglomeration effect; the H-H cluster is distributed around Shijiazhuang and Tangshan, and the L-L cluster is distributed in the middle region of Hebei.

Finally, the CCD between urbanization and urban resilience has a geographical spillover effect. With each 1% increase in the local coupling coordination degree, the neighboring counties will increase by 0.157% on average. The urban shape index, the government financial expenditure, the industrial upgrading level and the innovation level all have a considerable beneficial influence on the CCD, while the urban compactness has a significant negative impact on it.

The research findings may be used to guide the long-term development of new-type urbanization at the county level, as well as the improvement of the urban security in Hebei Province. It is critical to construct secure and resilient cities as part of the process of fostering the new-type urbanization strategies with county areas as the primary carriers. The government should optimize the urban spatial form, increase the government’s financial input in the field of public management, pay attention to industrial upgrading and scientific and technological innovation, implement city-specific policies and regional planning, to achieve a coordinated urbanization and urban resilience development.

## Figures and Tables

**Table 1:** Previous study of evaluation index system for urbanization.

Subsystem	Evaluation Indicators	Sources
Population urbanization	Percentage of non-agricultural population, proportion of employment in secondary and tertiary industries	Wang [64], Li [58], Liu [59], Shang [65], Liu [66]
Spatial urbanization	Urban population density, urban construction land (per 10,000 people), proportion of districts constructed in urban areas, transportation network density	Li [58], Liu [59], Lv [13], Long [67]
Economic urbanization	GDP, GDP per capita, proportion of the added value of the second and tertiary industry to GDP, gross industrial output value per capita, local fiscal revenue per capita, total fixed asset investment	He [57], Liu [59], Shang [65]
Social urbanization	Total retail sales of consumer goods per capita, consumption level of the residents per capita, number of phones (per 10,000 households), number of internet users (per 10,000 people), number of doctors (per 10,000 people), per capita disposable income of urban residents, people with a university degree, public library collections (per 10,000 people), number of social welfare adoption units (per 10,000 people)	Wang [64], He [57], Li [58], Liu [59], Lu [68], Shang [65]

**Table 2:** Previous study of evaluation index system for urban resilience.

Subsystem	Evaluation Indicators	Sources
Economic resilience	GDP per capita, GDP growth rate, amount of foreign investment utilized per capita, employment rate, energy intensity, tertiary industry as percentage to GDP, scientific research fund intensity, urban industrial innovation index, ratio of large-to-small businesses, large retail stores (per 10,000 people)	Cutter [61], Chu [69], Hu [27], Chen [49], Zhao [63]
Ecological resilience	Green coverage rate of built-up area, area of public green land per capita, NDVI, ecosystem stress index; air quality–excellent days ratio, ratio of centralized wastewater treatment, ratio of industrial solid wastes that are comprehensively utilized, mean annual concentration of PM_2.5_, carbon emission, land in wetlands, biodiversity, average percent perviousness	Cutter [61], Zhao [63], Kammouh [70], Delgado [53], Abdrabo [71]
Social resilience	Population with college education, population below 65 years of age, urban disposable income per capita, living area of urban residents per capita, urban population density, proportion of expenditure for social security and employment in fiscal expenditure, urban registered unemployment rate, urban Engel coefficient, number of undergraduates in regular HEIs, psychosocial facilities (per 10,000 people), families that provide telephone service, families with at least one car, community values cohesion	Cutter [61], Zhao [63], Cutter [60]
Infrastructure resilience	Road surface area per capita, number of buses owned (per 10,000 people), density of water supply pipelines in built-up area, density of sewers in built-up area, number of internet services (per 100 people), number of mobile telephones (per 100 people), night light, emergency shelter area per capita, housing stock construction quality, lifelines and critical infrastructure	Kammouh [70], Zhao [63], Cutter [61], Qiang [72]
Institutional resilience	Urban household registered population as percentage of total population, proportion of joining urban basic pension insurance for employees, fiscal expenditure per capita, urban maintenance and construction funds per capita, proportion of employees of enterprises, institutions and government in population, unit density of hospitals and health centers, number of doctors (per 10,000 people), performance regimes-state capital	Cutter [61], Zhao [63], Hu [27]

**Table 3:** Evaluation index system for urbanization and urban resilience.

Indicator Type	Subsystem	Evaluation Indicator	Meaning	Nature
Urbanization	Economic urbanization	A1	total amount of urban economic production	+
		A2	ratio of non-agricultural production output	+
	Population urbanization	A3	ratio of non-farm workers	+
		A4	population urbanization rate	+
	Spatial urbanization	A5	urban space expansion	+
		A6	per capita urban space resources	+
	Social urbanization	A7	social welfare level	+
		A8	social consumption level	+
Urban resilience	Economic resilience	B1	economic development level	+
		B2	level of economic growth	+
		B3	economic recovery capability	+
		B4	industrial production capacity	+
	Ecological resilience	B5	pre-disaster mitigation capabilities of ecosystems	+
		B6	green industrial development level	−
		B7		−
		B8	disaster reduction capacity of urban ecosystem	+
	Social resilience	B9	the human loss that may result from the disaster	−
		B10	disaster preparedness of the education system	+
		B11	disaster response capabilities of health systems	+
	Infrastructure resilience	B12	disaster-carrying capacity of urban space	+
		B13	post-disaster response capability of infrastructure	+
		B14	disaster-response capability of communication technology	+

“+” means positive to urban resilience, “−” means negative to urban resilience.

**Table 4:** Selection of driving factors.

Indicator Type	Evaluation Indicator	Meaning
Natural factors	RDLS	The relative height difference of the terrain within the county unit
Spatial factors	AWMSI	To measure the shape complexity of built-up areas, urban irregular sprawl typically raises AWMSI
	BCI	To measure the form compactness of built-up areas, a higher BCI suggests a more compact urban structure
Governance	ZC	The higher the public expenditure, the stronger the governance capacity of the government
Economy	CY	The higher the industrial output value ratio is, the stronger the industrial advanced degree is
Innovation	CX	The newer the scientific and technological enterprises are, the stronger the technological innovation capacity is

**Table 5:** Classification of the coupled coordination degree.

Classification	Criteria	Sub-Classification	Systematic Comparison
High coordination (I)	[0.8, 1]	High coordination (I-1)	0 ≤ |U2 − U1| ≤ 0.1
		Lagging urban resilience (I-2)	U1 − U2 > 0.1
		Lagging urbanization (I-3)	U2 − U1 > 0.1
Moderate coordination (II)	[0.7, 0.8)	Moderate coordination (II-1)	0 ≤ |U2 − U1| ≤ 0.1
		Lagging urban resilience (II-2)	U1 − U2 > 0.1
		Lagging urbanization (II-3)	U2 − U1 > 0.1
Mild coordination (III)	[0.6, 0.7)	Mild coordination (III-1)	0 ≤ |U2 − U1| ≤ 0.1
		Lagging urban resilience (III-2)	U1 − U2 > 0.1
		Lagging urbanization (III-3)	U2 − U1 > 0.1
Mild imbalance (IV)	[0.5, 0.6)	Mild imbalance (IV-1)	0 ≤ |U2 − U1| ≤ 0.1
		Lagging urban resilience (IV-2)	U1 − U2 > 0.1
		Lagging urbanization (IV-3)	U2 − U1 > 0.1
Moderate imbalance (V)	[0.4, 0.5)	Moderate imbalance (V-1)	0 ≤ |U2 − U1| ≤ 0.1
		Lagging urban resilience (V-2)	U1 − U2 > 0.1
		Lagging urbanization (V-3)	U2 − U1 > 0.1
Serious imbalance (VI)	[0, 0.4)	Serious imbalance (VI-1)	0 ≤ |U2 − U1| ≤ 0.1
		Lagging urban resilience (VI-2)	U1 − U2 > 0.1
		Lagging urbanization (VI-3)	U2 − U1 > 0.1

**Table 6:** *Moran’s I* of the coupling coordination degree.

Coupling Coordination Degree	*Moran I*	*Z* Score
CCD_2010	0.397 ***	7.997
CCD_2015	0.429 ***	8.623
CCD_2020	0.442 ***	8.924

*** *p* < 0.01.

**Table 7:** LM, LR test and Hausman of the spatial models with W1 and W2.

Test	LM Spatial Lag	Robust LM Spatial Lag	LM Spatial Error	Robust LM Spatial Error	LR Test-Spatial Lag	LR Test-Spatial Error	Hausman
W1	38.187 ***	20.643 ***	18.884 ***	1.341	42.94 ***	22.32 ***	20.73 ***
W2	85.643 ***	20.131 ***	156.421 ***	90.909 ***	154.81 ***	32.71 ***	30.67 ***

*** *p* < 0.01.

**Table 8:** Spatial Durbin model results with W1.

Model W1	(1) RE	(2) FE	(3) FEboth
Main			
RDLS	−0.027 (−1.147)	−0.035 (−0.680)	−0.037 (−0.753)
AWMSI	0.036 *** (2.973)	0.033 *** (7.600)	0.023 *** (5.950)
BCI	−0.019 ** (−2.229)	−0.020 ** (−2.325)	−0.018 *** (−4.028)
ZC	0.100 *** (5.514)	0.099 *** (2.689)	0.077 ** (2.131)
CY	0.052 ** (2.280)	0.055 *** (14.913)	0.044 *** (7.326)
KJ	0.042 *** (2.831)	0.042 * (1.811)	0.047 ** (2.168)
_cons	0.274 *** (8.610)	0.323 *** (4.994)	0.413 *** (9.666)
Wx			
RDLS	0.013 (0.249)	0.026 (0.615)	0.016 (0.506)
AWMSI	−0.081 **(−2.283)	−0.087 *** (−6.646)	−0.100 *** (−7.011)
BCI	0.017(0.749)	0.017 (0.361)	0.012 (0.321)
ZC	0.137 *** (4.532)	0.149 *** (6.051)	0.038 ** (2.031)
CY	0.068 * (1.890)	0.072 *** (6.483)	0.025 (1.247)
KJ	−0.001 (−0.025)	0.001 (0.045)	0.022 (1.477)
Spatial			
rho	0.355 *** (7.281)	0.320 *** (6.667)	0.157 *** (3.232)

*t* statistics in parentheses. * *p* < 0.1, ** *p* < 0.05, *** *p* < 0.01.

**Table 9:** Spatial Durbin model results with *W2*.

Model W2	(1) RE	(2) FE	(3) FEboth
Main			
RDLS	−0.000 (−0.600)	−0.003 *** (−3.648)	−0.003 *** (−3.921)
AWMSI	0.072 *** (4.064)	0.031 ** (2.068)	0.024 * (1.652)
BCI	−0.000 (−0.010)	−0.010 (−1.090)	−0.010 (−1.059)
ZC	0.096 *** (5.959)	0.098 *** (7.646)	0.082 *** (6.418)
CY	0.040 *** (2.652)	0.040 *** (3.442)	0.035 *** (3.001)
KJ	0.036 ** (2.510)	0.031 *** (2.782)	0.031 *** (2.870)
Wx			
RDLS	0.000 * (1.909)	0.006 *** (5.029)	0.004 *** (3.255)
AWMSI	0.069 * (1.917)	−0.094 *** (−2.833)	−0.105 *** (−3.219)
BCI	0.088 *** (3.697)	0.023 (1.168)	0.016 (0.822)
ZC	0.068 *** (2.693)	0.111 *** (4.709)	0.035 ** (1.287)
CY	0.004 (0.117)	0.040 (1.581)	0.020 (0.782)
KJ	−0.027 (−0.897)	0.026 (1.064)	0.026 (1.103)
Spatial			
rho	0.639 *** (17.202)	0.393 *** (7.583)	0.224 *** (3.532)

*t* statistics in parentheses. * *p* < 0.1, ** *p* < 0.05, *** *p* < 0.01.

## Data Availability

The data presented in this study are available on request from the corresponding author.
